# Evaluation of sample preparation methods for NMR-based metabolomics of cow milk

**DOI:** 10.1016/j.heliyon.2018.e00856

**Published:** 2018-10-19

**Authors:** Bénédict Yanibada, Hamid Boudra, Laurent Debrauwer, Cécile Martin, Diego P. Morgavi, Cécile Canlet

**Affiliations:** a Université Clermont Auvergne, INRA, VetAgro Sup, UMR Herbivores, F-63122, Saint-Genès-Champanelle, France; b Toxalim, Research Centre in Food Toxicology, Université de Toulouse, INRA, ENVT, INP-Purpan, UPS, F-31027, Toulouse, France; c Axiom Platform, MetaToul-MetaboHUB, National Infrastructure for Metabolomics and Fluxomics, F-31027, Toulouse, France

**Keywords:** Nutrition, Food analysis, Analytical chemistry

## Abstract

The quality of milk metabolome analyzed by nuclear magnetic resonance (NMR) is greatly influenced by the way samples are prepared. Although this analytical method is increasingly used to study milk metabolites, a thorough examination of available sample preparation protocols for milk has not been reported yet. We evaluated the performance of eight milk preparation methods namely (1) raw milk without any processing; (2) skimmed milk; (3) ultrafiltered milk; (4) skimming followed by ultrafiltration; (5) ultracentrifuged milk; (6) methanol; (7) dichloromethane; and (8) methanol/dichloromethane, in terms of spectra quality, repeatability, signal-to-noise ratio, extraction efficiency and yield criteria. A pooled sample of milk was used for all protocols. Skimming, ultracentrifugation and unprocessed milk protocols showed poor NMR spectra quality. Protocols involving multiple steps, namely methanol/dichloromethane extraction, and skimming followed by ultrafiltration produced inadequate results for signal-to-noise ratio parameter. Methanol and skimming associated to ultrafiltration provided good repeatability results compared to the other protocols. Chemical-based sample preparation protocols, particularly methanol, showed more efficient metabolite extraction compared to physical preparation methods. When considering all evaluation parameters, the methanol extraction protocol proved to be the best method. As a proof of utility, methanol protocol was then applied to milk samples from dairy cows fed a diet with or without a feed additive, showing a clear separation between the two groups of cows.

## 1 Introduction

Information on milk composition is used in the dairy industry to assess milk quality and to optimize technological processes. Milk is an attractive matrix for diagnostic purposes and to improve the farming system because of the presence of different classes of molecules. The non-invasive character of sample collection is also an advantage of this matrix (Jenkins et al., 2015). Analytical techniques like Mid-Infrared (MIR) spectroscopy (Ye et al., 2016) are routinely used to measure milk's major compounds like lipids, proteins, lactose (Di Marzo and Barbano, 2016) and minerals (Visentin et al., 2016). MIR spectroscopy shows benefits like rapidness and reproducibility but accurate identification of compounds is limited. Analytical techniques such as Nuclear Magnetic Resonance (NMR) and Mass spectrometry (MS) used in metabolomics are attractive because they provide both qualitative and quantitative information on hundreds of compounds present in biological matrices (de Graaf et al., 2015; Pinto et al., 2015). The information provided by such milk metabolomics studies has been applied to certify both geographical and species origins (Andreotti et al. 2000, 2002; Li et al., 2017) and to assess its technological and nutritional value (Sundekilde et al., 2011). Other studies investigated the physiology of lactation (Klein et al., 2010; Ilves et al., 2012) or methane mitigation strategies (Antunes-Fernandes et al., 2016) in dairy cows.

In a metabolomic study, each step is crucial to produce good quality data, starting from a standardized sample collection up to the analysis (Álvarez-Sánchez et al., 2010). Several papers showed that sample preparation strongly affects metabolite profile (Le Gall, 2015), highlighting the importance to optimize the process. A sample preparation method for global metabolomics analysis has to be non-selective, with a minimal number of steps (Vuckovic, 2012). Since each biological fluid has a particular composition, optimization is required for each analytical platform in order to produce optimal results. For NMR analysis, a good sample preparation method should generate reproducible spectra with low intersample chemical shift variability, provide high signal-to-noise ratio (SNR), and allow the detection of the highest number of metabolites. In addition, the method should preferably be rapid, robust, non-selective and compatible with high-throughput analyses. Several milk preparation methods have been reported in the literature. These methods are diverse and range from the use of unprocessed milk with simply the addition of deuterated water before analysis (Hu et al., 2007) to various physical and chemical processing methods such as centrifugation (skimmed milk) (Sundekilde et al., 2011; Tian et al., 2016), ultrafiltration (Klein et al. 2010, 2012), centrifugation and ultrafiltration (Sundekilde et al., 2013a), ultracentrifugation (Lu et al., 2013), methanol (protein precipitation) (Wu et al., 2016), and chloroform (Lamanna et al., 2011). Each method has advantages and disadvantages. Physical separation protocols do not affect the concentration of volatile compounds as there is no evaporation step involved. However, the recovery of some metabolites can be affected by the type and amount of macromolecules characteristic of milk from different mammals. In contrast, chemical precipitation protocols are useful to discard macromolecules such as proteins and lipoproteins. This is important for NMR metabolomics analysis since macromolecules can hinder metabolite signals and hamper quantification (Beckonert et al., 2007) but an evaporation step is necessary. Comparison of sample preparation protocols was reported for blood (Nagana Gowda et al., 2015), feces (Lamichhane et al., 2015) and tissues (Wu et al., 2008). For dairy milk, to the best of our knowledge, the comparison of the existing protocols for NMR analysis has not been reported. In the present study, we developed a systematic and standardized procedure to compare different milk sample preparation protocols for NMR metabolomics analysis. Various parameters were used, namely 1D NMR spectrum quality, signal-to-noise ratio (SNR), repeatability, efficiency and yield of extraction. Subsequently, the best protocol was then used to compare metabolite profile of milk samples obtained from dairy cows fed a diet with or without a feed additive.

## 2 Materials and methods

Milk samples used were obtained from previous animal studies conducted according to procedures approved by the regional ethical committee and in accordance with applicable French and European guidelines. The approval numbers are CE50-12 and 821-2015060811534198.

### 2.1 Chemicals

Methanol and dichloromethane were purchased from Scharlau SL (Sentmenat, Spain), deuterium oxide (D_2_O) and sodium 3-trimethylsilyl-2,2,3,3-tetradeuteriopropionate (TSP) from Eurisotop (Saint-Aubin, France). Hippuric acid, 3-hydroxybutyric acid, citric acid, formic acid, and L-Alanine were purchased from Sigma Chemical Company (Saint Louis, MO).

### 2.2 Sample preparation

Protocols were evaluated by analyzing replicate sets (n = 6) of quality control samples (QC) prepared from a pooled milk sample that were collected (10 ml from each cow) from four lactating Holstein cows (54 ± 14 days in milk) receiving a diet consisting of 56% corn silage, 4% hay and 40% of concentrate. Milk samples were collected at each milking, twice in a single day aliquoted by fraction of 500 μl and immediately stored at -80 °C. Samples were thawed on ice and mixed for each individual cow at a 3:2 ratio for the morning and evening milking, respectively. In this study, 8 different sample preparation protocols described in the literature were evaluated: (1) raw milk without any processing (Hu et al., 2007); (2) skimmed milk (Sundekilde et al., 2011), (3) ultrafiltered milk (Klein et al. 2010, 2012), (4) skimming followed by ultrafiltration (Sundekilde et al., 2013a), (5) ultracentrifuged milk (Lu et al., 2013); (6) methanol (Wu et al., 2016), (7) dichloromethane (Lu et al., 2015) and (8) methanol/dichloromethane (Pratico et al., 2014; Andreas et al., 2015). Protocols 2 to 5 are physical separation protocols using centrifugation and/or filtration whereas protocols 6 to 8 are based on chemical sample preparation. Samples were thawed on ice, homogenized by vortex mixing and processed following each specific procedure. To minimize changes in the metabolome during sample preparation, samples were kept on ice or at 4 °C whenever possible and each protocol was performed as quickly as possible, i.e. no waiting time between steps.

For physical separation protocols, skimmed milk samples were prepared by centrifugation of 500 μl of milk at 1,000 g for 15 min. The aqueous layer was aspirated and transferred into 1.5 ml Eppendorf tubes for a second centrifugation step using the same parameters. Ultrafiltration was undertaken with Amicon centrifugal filter units (cut-off 10 kDa) (Merck KGaA, Darmstadt, Germany) as described by (Sundekilde et al., 2013a). Filters were prewashed as described by (Zacharias et al., 2013). A double wash was necessary to remove contaminants and protective agents released by the filter. A preliminary test was performed on protocols using ultrafiltration to determine the minimum milk dilution that gave the largest volume of filtered milk. Different volumes of water (50-100-150-250 and 300 μl) were added into 500 μl milk, vortex mixed and centrifuged at 14,000 g for 20 min at 4 °C. Adding a volume of 50 μl of water was optimal for ultrafiltration protocol but none of the tested volumes changed the volume recovered from the protocol combining skimming and ultrafiltration, as the skimming steps discarded a great amount of high molecular weight compounds. Ultracentrifugation was performed at 140,000g for 75 min at 4 °C (Beckman L8-55M, Brea, USA). The lower layer was collected in glass tubes, centrifuged at 16,000 g for 20 min at ambient temperature (20 °C) to remove remaining fat and the supernatant was used for analysis. For each physical separation protocol, the final volume collected was measured and adjusted to 500 μl with Milli-Q water.

For chemical based preparation protocols, 500 μl of milk samples were transferred into Pyrex glass tubes to avoid any interaction between the solvent and the container. For the methanol protocol as described by Wu et al. (2016), 1 ml of cold methanol was added to the milk sample. The mixture was vortexed, stored at -20 °C for 30 min and centrifuged at 18,000 g for 10 min at 4 °C. The methanol-water solution was then transferred to a glass tube using a Pasteur pipette and evaporated to dryness using a Speedvac evaporator (Thermo Scientific, Waltham, USA) without heating for 3 hours. The residue was dissolved in 500 μl of Milli-Q water for the analysis of polar compounds. The dichloromethane protocol was described by Lu et al. (2015). Briefly 1 ml dichloromethane was mixed with 500 μl of milk, vortexed for 20 seconds, and centrifuged at 8,000 g for 20 min at room temperature (20 °C). The milk aqueous layer was collected, the volume was adjusted to 500 μl and analyzed without any other processing. The methanol/dichloromethane protocol (Pratico et al., 2014; Andreas et al., 2015) involved the use of 6 ml of methanol/dichloromethane mixture (1:2, v: v). Samples were homogenized with a vortex mixer for 10 seconds and centrifuged at 1,500 g for 5 min at 4 °C. The supernatant was collected (2.3 ml), mixed with 1.2 ml of NaCl (0.9%), and centrifuged as described in the first step. The top aqueous layer was collected, evaporated to dryness as described above for the methanol protocol and the residue was dissolved in 500 μl of Milli-Q water for the analysis of polar compounds.

Metabolite quantification and recovery were tested on protocols that passed the first NMR spectra quality step. Samples were spiked at 0.12 M with a standard mixture containing hippurate, 3- hydroxybutyrate, alanine, citrate, and formate. These five metabolites were chosen as they are naturally present in milk and they display chemical shifts covering a broad spectrum range (from 1.47 to 8.46 ppm). For each protocol, three replicates were spiked with the standard mixture and three without as follows: to 250 μl of QC sample, 50 μl of standard mixture or 50 μl of Milli-Q water were added. After the different sample preparations, Milli-Q water was added if necessary to obtain a final volume of 500 μl. Control samples were also prepared, using 50 μl of the standard mixture and 450 μl of Milli-Q water.

The chosen protocol was finally tested using samples from an independent study to validate the methodology. For that, we used milk samples obtained from individual cows from a study described by (Guyader et al., 2016). In this study, lactating cows were allocated into two groups that were fed a similar diet consisting of 54% maize silage, 6% of hay, and 40% of concentrate supplemented (Treated, n = 8) or not (Control, n = 8) with 3.5% fat from linseed and 1.8% nitrate. Linseed and nitrate are known to reduce methane emission without affecting digestibility and animal health (Doreau et al., 2014). Both groups were balanced for calving date, milk production and had similar proportion of crude protein, starch, and fiber content in the diet. All these factors are known to influence milk composition and milk metabolome (Antunes-Fernandes et al., 2016). We choose this study because we hypothesized that differences in milk metabolome between the two groups would not be large and to be able to detect these differences using a relatively reduced number of individuals the analytical variance accounted for the sample preparation protocol needs to be low. Milk samples were collected in week 16 of the study, stored at – 80 °C and processed as described above.

### 2.3 NMR analysis

Before NMR analysis, 200 μl of deuterium oxide (D_2_O) phosphate buffer (pH 7) solution containing sodium trimethylsilyl propionate (TSP) was added to milk samples to avoid possible chemical shift drifts due to pH effects (De Marco, 1977). Samples were then vortexed and centrifuged at 5,500 g for 10 min at 4 °C and the supernatants were analyzed by 1H NMR spectroscopy. The supernatants (600 μl) of each sample were transferred into 5 mm NMR tubes. ^1^H NMR spectra were acquired on a Bruker Avance III HD spectrometer (Bruker, GMBH, Karlsruhe, Germany) operating at 600.13 MHz, equipped with an inverse detection 5 mm ^1^H-^13^C-^15^N-^31^P cryoprobe connected to a cryoplatform and a cooled SampleJet sample changer, using TopSpin 3.2 software (Bruker, GMBH, Karlsruhe, Germany). The temperature was allowed to stabilize for 5 min after insertion into the magnet. Tuning, matching, and shimming was performed for each sample, and the ^1^H pulse length was calibrated on each sample and was typically around 10 μs. Spectra were acquired using a Carr-Purcell-Meiboom-Gill (CPMG) spin echo sequence used to reduce macromolecular signals (Gowda et al., 2008) with a 5 seconds-relaxation delay to attenuate proteins and lipoproteins broad signals. A water suppression signal was achieved, by presaturation during the relaxation delay. The spectral width was set to 20 ppm for each spectrum and 128 scans (16 dummy scans) were collected with 32K points. Free induction decays were multiplied by an exponential window function (LB = 0.3 Hertz used to avoid broad signals) before Fourier Transform. The spectra were manually phased and the baseline was corrected. All spectra were referenced to TSP signal at 0 ppm. A 1D NOESY pulse sequence was used to evaluate amounts of residual macromolecule signals. A relaxation delay of 5 seconds was used, the receiver gain was set to 45.2 and 128 scans with 32 K points were collected. Also, zgpr 1D pulse sequence was used for quantitative NMR. A relaxation delay of 8 seconds was used, the receiver gain (rg) was set to 36, and 512 scans were collected with 32K points. The line broadening was set at 0.3.

### 2.4 Data quality assessment

Before analysis of milk samples, sensitivity, lineshape, and water suppression were checked using NMR sample reference tubes 0.1% Ethylbenzene in CDCl_3_ (Bruker, Z10121), 1% CHCl_3_ in acetone-*d*_*6*_ (Bruker, Z10250), and 2mM sucrose 10% D_2_O (Bruker, Z10268), respectively. After shimming optimization, values obtained corresponded to manufacturer's specifications. Temperature stability was checked with the 4% Methanol in methanol-*d*_*4*_ NMR standard tube (Bruker, Z10128). The shim quality was appraised using the line width of TSP at 50% of its full height (0.86 Hz), as it is recommended by (Sumner et al., 2007). The line width at half height of residual water was 2.72 Hz indicating that the water suppression was within specification. The baseline was manually corrected, removing distortions and setting the corrected baseline near to the zero intensity level. Aware that TSP might interact with proteins in milk, we verified that TSP did not have any negative effect on calibration and normalization by following the recommendations made by (Beckonert et al., 2007). In order to detect eventual protein binding on TSP, the measurement of the width at half height for each spectra was done. TSP signal was considered good if the value was lower than 2.5 (with a line broadening of 1 Hz). The line width half height is inversely proportional to the relaxation time. Since macromolecules such as proteins have small relaxation times, TSP binding to proteins would lead to line width half heights larger than 10 Hz when using a CPMG sequence. We evaluated the amount of residual macromolecules in different sample preparations. The superposition of zgpr and CPMG spectra helped to identify attenuated signals belonging to macromolecules. The calculation of integral signals and the comparison to the spectra integral (without water signals) gave an estimation of the extent of residual macromolecule signals. Resolution was quantified by calculating, width at half height for each signal.

### 2.5 Data processing and statistical analysis

The NMR spectra were imported in the Amix software (version 3.9, Bruker, Rheinstetten, Germany) for data processing. A variable size bucketing was used based on graphical pattern (166 buckets), each integrated region was normalized to the TSP signal. Data were analyzed by chemometrics tools as unsupervised principle component analysis (PCA) and supervised orthogonal projections to latent structures-discriminant analysis (OPLS-DA) (Trygg and Wold, 2002) using SIMCA P+ (V14, Umetrics AB, Umea, Sweden). PCA was first performed to look for trends, clusters between protocols or identify potential outliers. For the samples used for validation, based on both Hotelling and the distance to the model (DModx) plots, two samples from the treated group were outliers and then removed. These two samples had altered spectra with an important methanol signal, probably caused by incomplete evaporation of methanol. Supervised OPLS-DA was performed on validation samples to reveal potential markers of response to additive supplementation. The performances of the models were assessed by their predictive ability (Q^2^) and the proportion of explained variable (R^2^). A cross-validation procedure was used to assess the robustness of the model using the permutation test proposed by SIMCA P+ (V14, Umetrics AB, Umea, Sweden). Most predicted values (Q^2^) obtained by the permutation test were equal or lower than those of the original model indicating its validity (Fig. 5B).

### 2.6 Performance evaluation

For each protocol, spectrum quality, SNR of each signal, intra-run variability and sample preparation efficiency were checked. For spectrum quality, signals shapes were visually evaluated. For protocols that successfully pass this first evaluation, they were evaluated in terms of signal to noise ratio (SNR), repeatability, extraction efficiency. The SNR was evaluated by designing spectral region integral for each sample preparation methods, to define signal and noise (10–10.5 ppm) regions for the SNR calculation. Topspin “Sino” function based on an automatic calculation of the most intense SNR for each defined region was applied. The threshold of SNR to consider a peak as a real signal was set to 20. The repeatability was evaluated on the selected buckets (n = 166). The repeatability between the different protocols was first visualized using PCA (see Supplementary Material S1) (Trygg et al., 2007; Lindon et al., 2001), and then evaluated by calculating the coefficient of variation (CV) defined as the ratio of the standard deviation to the mean of the signal intensities, which was considered as acceptable if lower than 10%. The extraction efficiency was assessed based on a geometric method proposed by (Martineau et al., 2011), it was assessed by evaluating the contribution of each bucket in the discrimination process among the protocols. Also, the concentration of 10 identified metabolites namely butyrate, valine, isoleucine, acetone, glutamate, citrate, creatine, galactose, orotate and hippurate was determined in different protocols. The calculation was performed using TSP integration value set to an arbitrary value of 100. The calculation used signal multiplicity, integration value and TSP concentration. Additionally, metabolite recovery was tested with QC samples spiked with a standard mixture containing five metabolites (see Supplementary Material S2). NMR spectra obtained on unspiked and spiked QC and the pure solution of standards were compared.

### 2.7 Metabolite annotation

The annotation of metabolites was performed, in the first instance by comparing chemical shifts and coupling constants, with ^1^H NMR spectra of reference compounds acquired under the same conditions. We further followed the annotation by comparing our data with published studies (Sundekilde et al., 2013a,b; Yang et al., 2016; Li et al., 2017; Klein et al., 2010) and on standards implemented in databases like the Human Metabolome Database (Wishart et al., 2007) (http://www.hmdb.ca/metabolites) and the Biological Magnetic Resonance Data Bank (Ulrich et al., 2008) (http://www.bmrb.wisc.edu/metabolomics/metabolomicsstandards.shtml). Identified chemical structures were confirmed using two-dimensional NMR experiments (2D J-Resolved 1H NMR spectroscopy, 2D ^1^H-^1^H Correlation Spectroscopy, 2D ^1^H-^13^C Heteronuclear Single Quantum Spectroscopy). According to Sumner et al. (2007), metabolites are identified at the Metabolomics Standards Initiative (MSI) level 1 if two independent and orthogonal data are identical to those of a reference compound analyzed under the same conditions (for example ^1^H NMR spectrum and 2D ^1^H-^13^C HSQC NMR spectra). If this information is not available the metabolite is putatively annotated from publications or databases (MSI level 2). The identification MSI level is given for each metabolite.

## 3 Results

### 3.1 1D NMR spectra quality

**Fig. 1:**
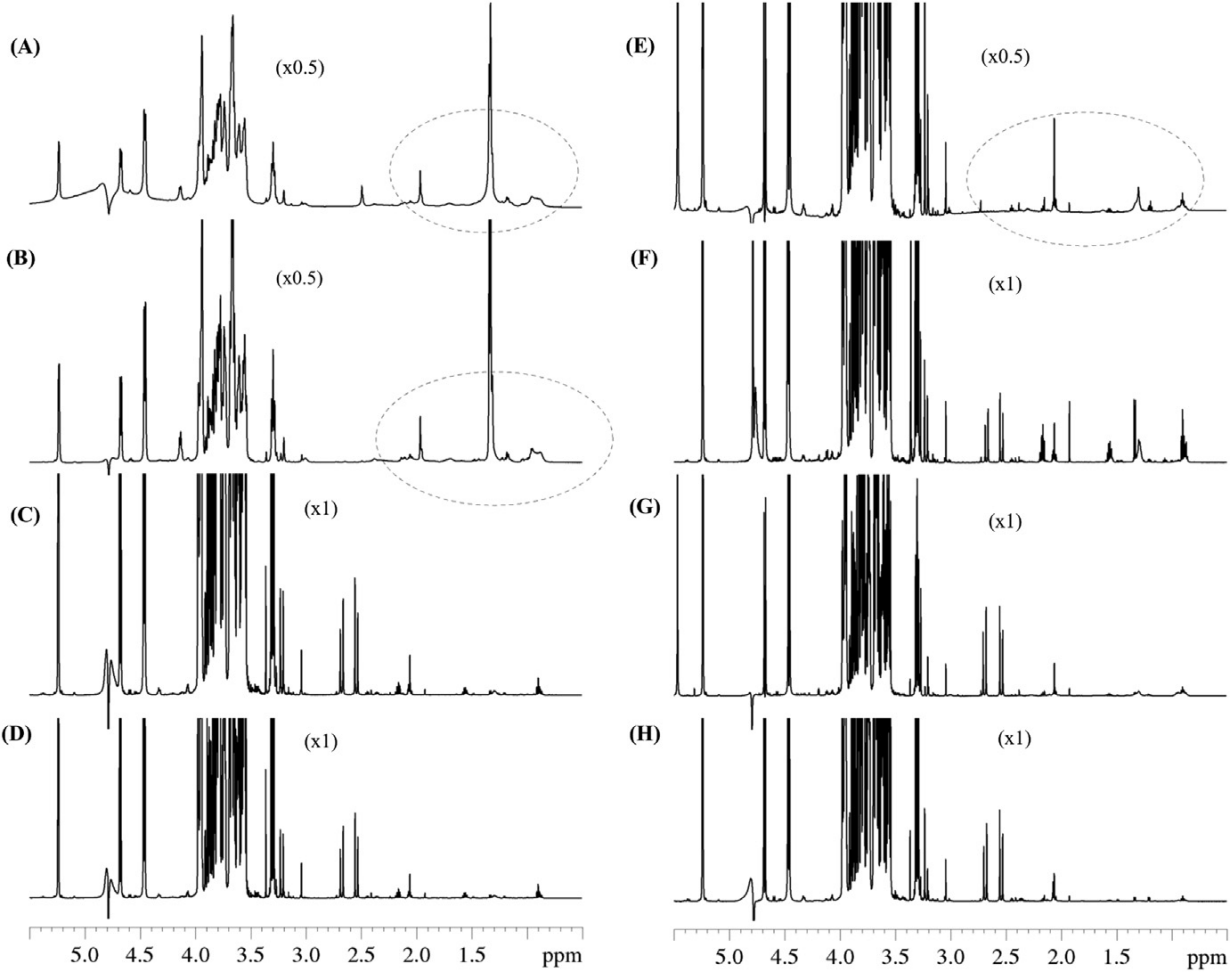
600 MHz 1D 1H NMR CPMG spectra obtained from cow milk prepared by different protocols. Raw milk without any processing (A), skimmed milk (B), ultrafiltered milk (C), skimming followed by ultrafiltration (D), ultracentrifuged milk (E), methanol (F), dichloromethane (G) and methanol/dichloromethane (H). Circles in the spectra indicates regions containing signals from macromolecules.

The 1D NMR spectra quality was compared for the different protocols. Analysis of raw milk without any processing, milk skimming and milk ultracentrifugation led to spectra displaying broad signals (Fig. 1A, B and E). For instance, spectra obtained without any processing or following skimming showed large signals from 0.84 to 2.5 ppm, as shown in Fig. 1. These signals likely correspond to macromolecules which were not efficiently removed and which may hinder resonance signals from relevant metabolites. In contrast, ultrafiltration or methanol protocols (Fig. 1C and F) provided accurate and well resolved signals. On the basis of this first observation, the protocol without any processing as well as skimming and ultracentrifugation protocols were excluded for the next steps of the evaluation. For the five other protocols, the amount of residual macromolecules were quantified. Physical extraction protocols had the lowest amount of residual macromolecular signals compared to protocols with a precipitation step, particularly dichloromethane. The 1D NOESY sequence shows that residual macromolecules signals was 2.10% for dichloromethane, 0.40% for methanol, 0.85% for methanol/dichloromethane, 0.03% for ultrafiltration and 0.04% for skimming associated with ultrafiltration. The comparison of 1D NOESY, zgpr and CPMG sequences for each protocol confirms that CPMG attenuate macromolecule signals (Data are available on the Metabolights database).

### 3.2 Signal-to-noise ratio

The remaining five protocols were then assessed on the basis of SNR. A total of 368 signals could be observed along the spectral region. Table 1 indicates that the dichloromethane protocol yielded the highest percentage of buckets displaying a SNR greater than 20, followed by methanol and ultrafiltration protocols that gave similar percentages. Protocols involving multiple steps, namely methanol/dichloromethane and skimming and ultrafiltration led to lower SNR values. The dichloromethane protocol had a high width at half height value for the reference compound (TSP), revealing the presence of contaminating proteins (Table 1). In contrast, the other protocols had values lower than 2.5 Hz. Width at half height was also determined for signals with a SNR ratio higher than 20. The results show that physical based protocols had better results in terms of number of signal with low width at half height value, meaning a better resolution (Table 1).

**Table 1:** Sample preparation methods percentage of signal to noise ratio over 20 (368 signals) and signals wide at half height.

Protocols	Signals^1^ with SNR (%) > 20	TSP average wide at half height (Hz)^2^	% of signals with a wide at half height higher than 2.5 Hz
Ultrafiltration	66	1.6 ± 0.2	12
Skimming/ultrafiltration	60.5	2.0 ± 0.6	19
Methanol	67.1	1.8 ± 0.1	46
Dichloromethane	72.5	5.9 ± 0.3	30
Methanol/dichloromethane	62.2	1.9 ± 0.5	40

1 The signal to noise calculation was performed using Top-spin “sino” function. A threshold of 20 was fixed.

2 TSP signal is considered good if the value was lower than 2.5 (with a line broadening of 1 Hz).

### 3.3 Repeatability tests

**Fig. 2:**
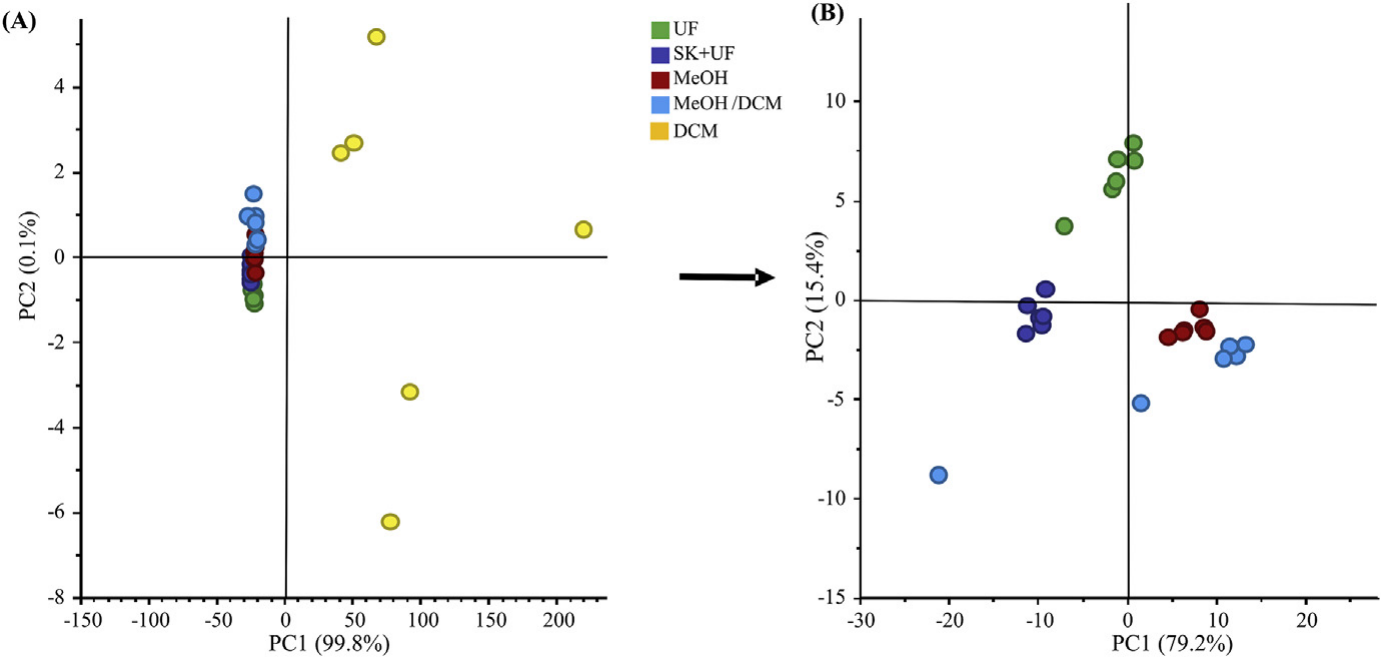
Principal Components Analysis score plot of the 1D 1H NMR CPMG spectra from cow milk prepared by **(A)**: Ultrafiltration (UF) (green circle); Skimming associated with ultrafiltration (SK + UF) (blue circle); Methanol (MeOH) (red circle); Dichloromethane (DCM) (yellow circle); Methanol/dichloromethane (MeOH/DCM) (cyan circle), and **(B)** after removing dichloromethane sample preparation. Dichloromethane (plot A) and MeOH/DCM (plot B) protocols show high variability; whereas remaining protocols were tightly clustered. The first two components explained more than 95% of the variance. Each dot in the score plot represents a NMR spectrum corresponding to a sample.

The PCA score plot clearly shows that samples processed by the dichloromethane protocol were loosely clustered and differed from the other protocols (Fig. 2A). When removing dichloromethane samples, the PCA score plot shows three tight clusters for ultrafiltration, skimming/ultrafiltration and methanol treated samples, and another cluster corresponding to methanol/dichloromethane samples that display variability (Fig. 2B). The CV calculated from buckets intensities was used to compare the protocols. For each method, buckets with a CV lower than 10% were considered as acceptable. Results indicate that methanol sample preparation provided the highest number of buckets (137/166) with a CV lower than 10%, followed by skimming associated with ultrafiltration (121/166) and ultrafiltration (108/166) protocols. In contrast, no single acceptable bucket was provided by dichloromethane and methanol/dichloromethane protocols. In agreement with the high width at half height reported above for TSP, this result confirms the presence of contaminating macromolecules in samples prepared with the dichloromethane protocol. High TSP wide at half height value and low repeatability are incompatible with quantitative analysis and, therefore, the dichloromethane protocol was discarded in subsequent steps.

### 3.4 Extraction efficiency

**Fig. 3:**
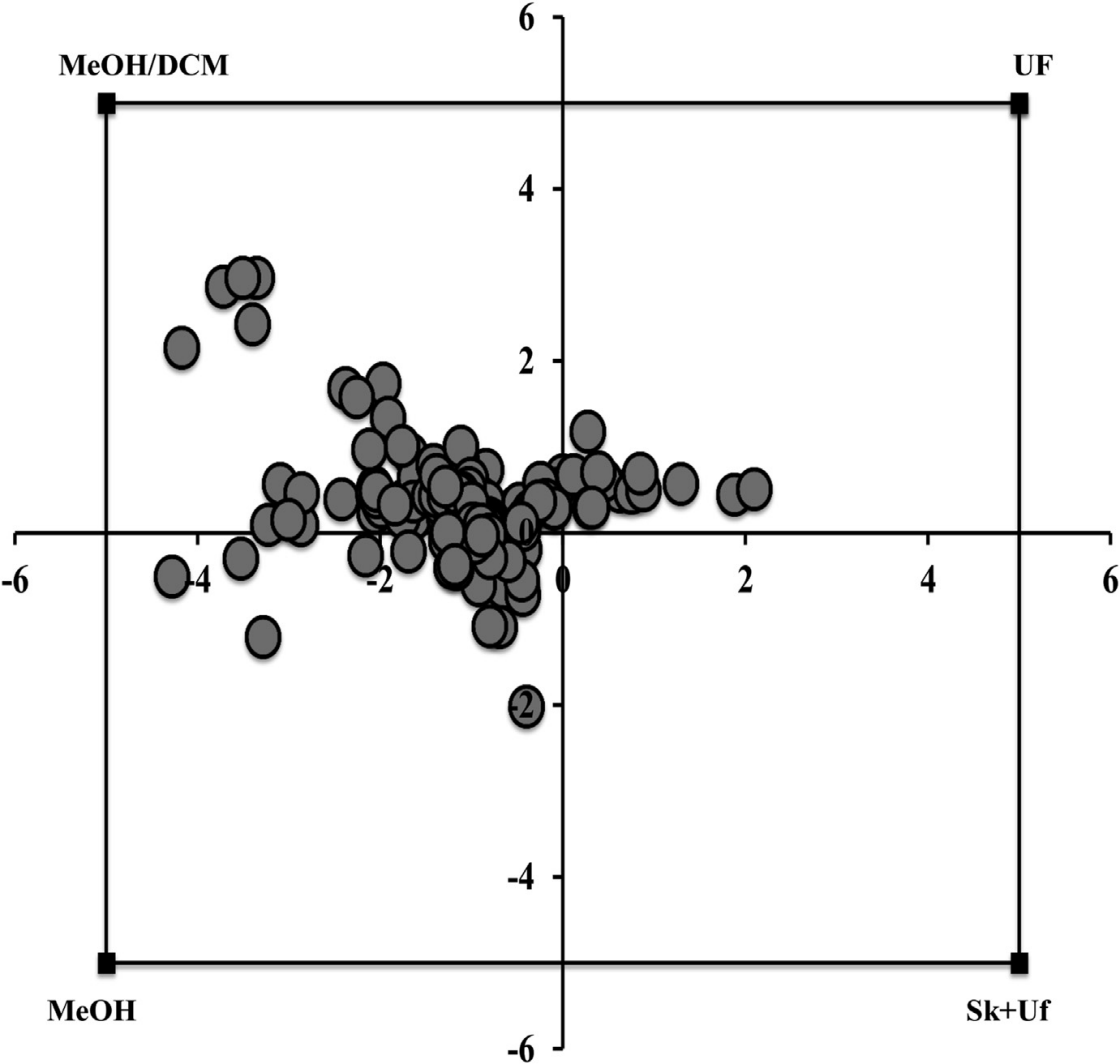
Graphical representation of the efficiency of sample preparation methods for cow milk. Buckets integration data were plotted on a square where each corner represents one of the four preselected sample preparation methods namely ultrafiltration (UF), skimming associated with ultrafiltration (SK + UF), methanol (MEOH) and methanol/dichloromethane (MEOH/DCM) sample preparation, and each bucket is represented by a dot. For protocols giving greater integration values, buckets are positioned close to the corresponding corner of the square, illustrating the efficiency of the corresponding technique to extract the metabolite. A bucket positioned in the center of the square has the same weight for the four sample preparations.

For the four remaining protocols, extraction efficiency was assessed based on a geometric method proposed by (Martineau et al., 2011), which allows to highlight differences in profiles induced by the various sample preparation. Fig. 3 shows that the two chemical protocols (methanol and methanol/dichloromethane) were the most efficient for extracting milk metabolites in terms of integration value. In addition, recovery rate calculation was determined on five spiked metabolites (Table 2). These metabolites which are commonly reported in milk analysis, are evenly spread over the spectral window and are accurately quantified without signal superposition. Methanol protocol gave the best results with yields ranging from 70% to 87%, followed by methanol/dichloromethane protocol with yields comprised between 65% and 77%. The two physical based separation protocols showed low recoveries for all metabolites with yields lower than 50%. Comparison of recoveries obtained using ultrafiltration with and without prior skimming indicates that the skimming step was responsible for a signal loss ranging from 6 to 16% depending on the considered metabolite.

**Table 2:** Recovery rate (%) of five metabolites spiked in milk obtained for the tested sample preparation methods.

Protocols	Recovery (mean ± SD, n = 3)			
	Ultrafiltration	Skimming/ultrafiltration	Methanol	Methanol/dichloromethane
Formate	51 ± 5	35 ± 7	83 ± 6	67 ± 6
Hippurate	39 ± 4	29 ± 6	76 ± 1	77 ± 7
Citrate	34 ± 3	28 ± 9	70 ± 7	65 ± 8
3-Hydroxybutyrate	48 ± 5	37 ± 7	87 ± 3	69 ± 6
Alanine	47 ± 5	35 ± 7	87 ± 6	72 ± 7

Metabolites chemical shifts: formate (8.46 ppm); hippurate (7.55 ppm - 7.64 ppm - 7.82 ppm); 3 hydroxybutyrate (4.15 ppm-2.40 ppm - 2.31 ppm-1.20 ppm); alanine (1.47 ppm-3.78 ppm); citrate (2.66 ppm-2.53 ppm).

**Fig. 4:**
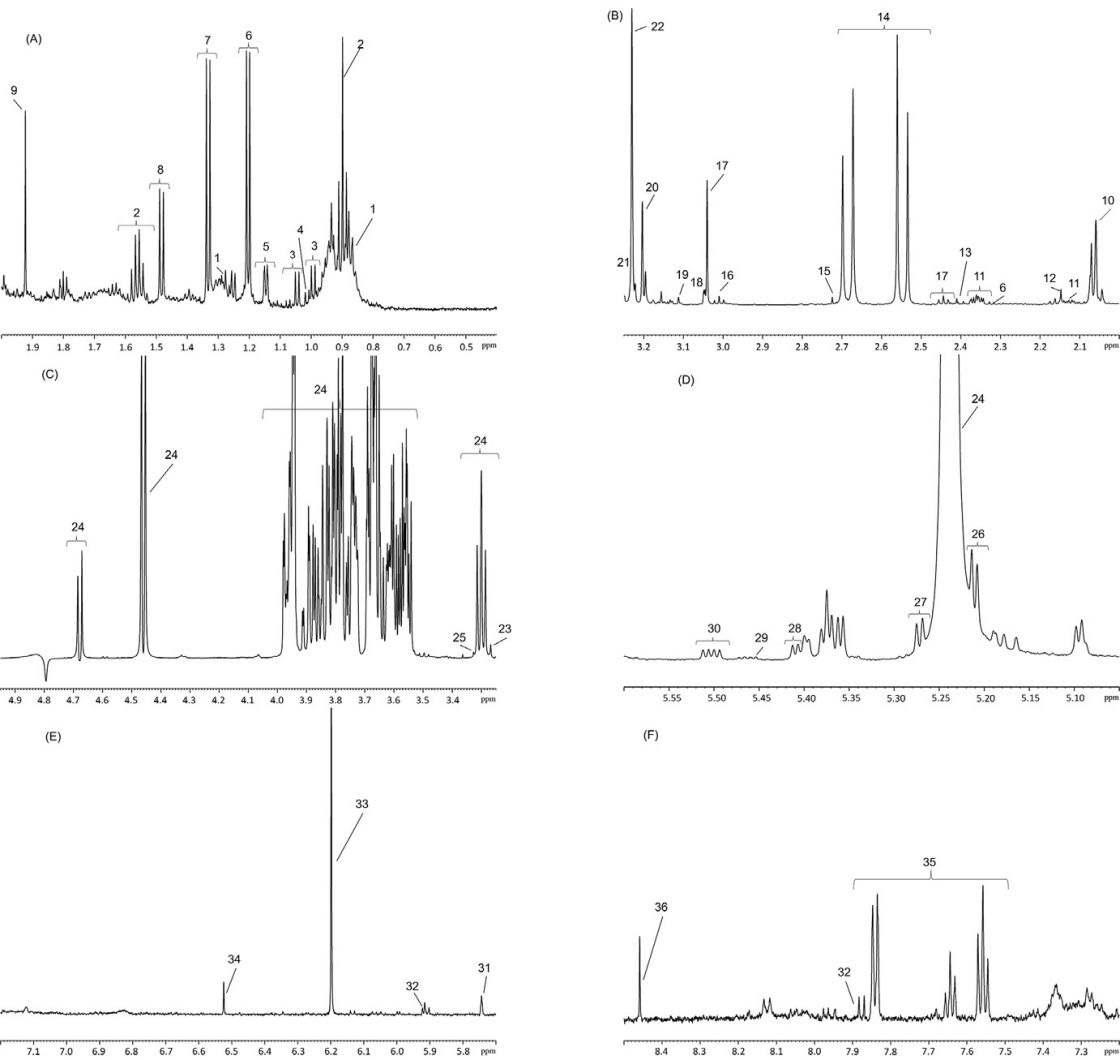
Typical NMR spectrum obtained from cow milk using the methanol protocol for sample preparation. The spectrum was divided into 6 parts: **(A) 1.** Caproate, **2.** Butyrate, **3.** Valine, **4.** Isoleucine, **5.** Propylene Glycol, **6.** 3-Hydroxybutyrate, **7.** Lactate; **8.** Alanine, and **9.** Acetate; **(B) 10.** N-Acetyl Glucosamine, **11.** Glutamate, **12.** Methionine, **13.** Succinate, **14.** Citrate, **15.** Dimethylamine; **16.** 2-Oxoglutarate, **17.** Creatine/Phosphocreatine, **18.** Creatinine, **19.** Dimethylsulfone, **20.** Choline/Phosphocholine, **21.** Glycerophospocholine, and **22.** Carnitine; **(C) 23.** Betaine, **24.** Lactose, and **25.** Methanol; **(D) 24.** Lactose, **26.** Mannose, **27.** Galactose, **28.** Maltose, **29.** Glucose-1-phosphate, **30.** UDP-N-acetyl glucosamine; **(E) 31.** Cis-aconitate, **32.** Uridine, **33.** Orotate, and **34.** Fumarate; **(F) 32.** Uridine, **35.** Hippurate, and **36.** Formate.

Based on all results, the methanol protocol was chosen for validation. Additionally, metabolite annotation was undertaken on the NMR spectra. Thirty-six metabolites, mainly amino acids, carbohydrates, and organic acids, were annotated (MSI level 1, n = 25) or putatively annotated (MSI level 2, n = 11) as shown in Fig. 4A–F (see Supplementary Material S3).

### 3.5 Application of the selected protocol on experimental data

**Fig. 5:**
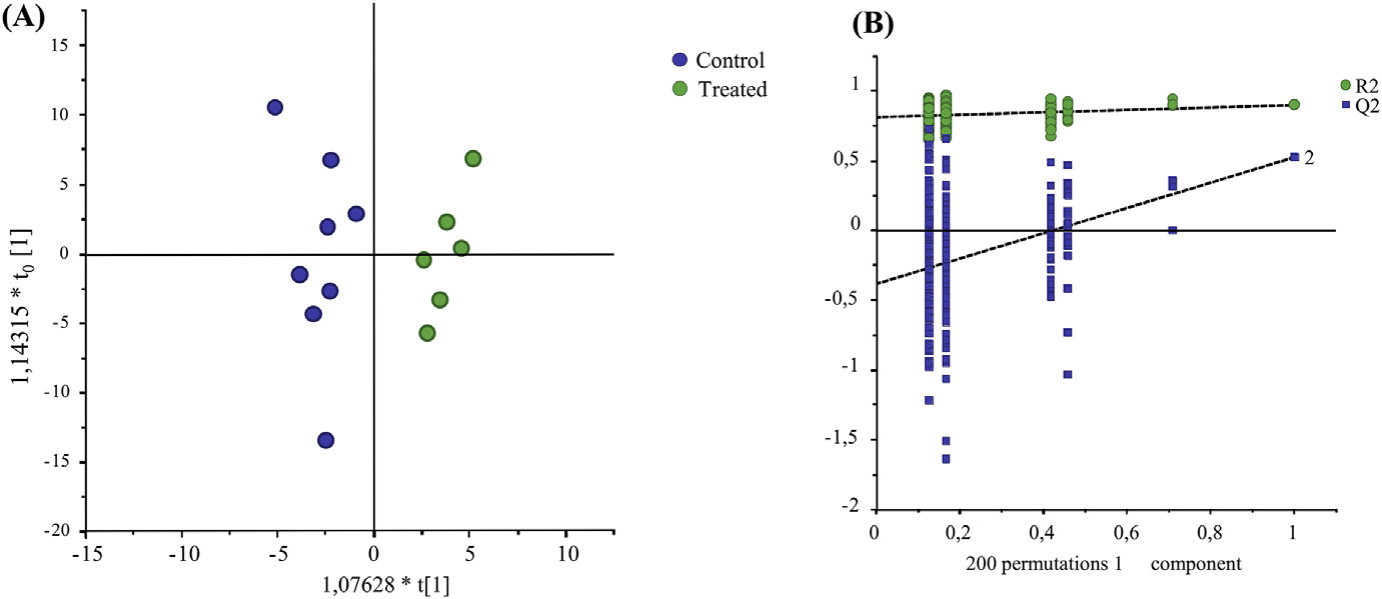
(**A**) Orthogonal projections to latent structures-discriminant analysis (OPLS-DA) score plot of the 1D 1H NMR CPMG spectra of methanol-processed milk samples from two groups of cows fed a similar diet supplemented or not with linseed oil-nitrate additive. Control (blue circles) and treated group (green circles) (R^2^X = 0.418, R^2^Y = 0.902, Q^2^ = 0.527), (2 samples were discarded as there were outliers in the PCA score plot). **(B)** Permutation test of the OPLS-DA model.

As part of the validation process and to confirm that the analytical variance was lower than the biological variance, the methanol sample preparation method was tested on experimental samples (data can be found in Supplementary Material S4). Orthogonal Partial Least Square Discriminant analysis shows a clear separation of milk samples according to groups (Fig. 5A). The model, validated by a permutation test, had a good explained variance (R2X = 0.418) and predictivity (Q^2^ 0.527). The permutation test confirmed the robustness of the model (Fig. 5B). More than 90 variables important in the projection (VIP) with a value higher than 1.3 were responsible for the differences between groups, among them hippuric acid, citric acid, betaine, orotic acid and glucose-1-phosphate were identified (Table 3).

**Table 3:** Milk metabolites identified in cows fed a similar diet supplemented (Treated) or not (Control) with linseed oil-nitrate additive.

Metabolites	VIP value^1^	Chemical shift (ppm)^2^	Identification MSI^3^	Fold change^4^
Hippurate	1.89	7.835 (t)	1	1.42
Citrate	1.93	2.705 (dd)	1	2.01
Betaine	1.86	3.265 (s)	1	1.20
Orotate	1.32	6.175 (s)	2	2.70
Glucose-1-phosphate	2.05	5.435 (dd)	2	1.88

1 The bucket with the highest VIP value is given.

2 Multiplicity: (dd) = doublet of doublet; (s) = singlet; (t) = triplet.

3 Level 1 (annotated): confirmed by two independent and orthogonal data relative to an authentic compound analyzed under identical experimental conditions, for example ^1^H NMR and 2D ^1^H-^13^C HSQC NMR spectroscopy. Level 2 (putatively annotated): Comparison with a standard using a single acquisition sequence, for example ^1^H NMR spectroscopy as recommended by the Metabolomics Standard Initiative (MSI) (Sumner et al., 2007).

4 Ratio of the mean intensity between Treated and Control replicates.

## 4 Discussion

Sample preparation is a crucial step for metabolomic analysis (Dunn and Ellis, 2005; Duportet et al., 2012). For NMR, sample preparation aims to extract the maximum number of metabolites without interfering macromolecules. Therefore, protocols involving a precipitation or ultrafiltration step are often used to discard macromolecules. Our results agree as raw milk analysis, skimming, ultracentrifugation and dichloromethane protocols, which do not remove macromolecules, were unsuitable. For other protocols, residual macromolecule signals were evaluated on spectra but their proportion was weak, less than 1% of the total intensity of the spectrum (Gowda and Raftery, 2014). Also, good TSP signals were obtained (between 1.6 Hz for ultrafiltration and 2.0 Hz for skimming associated with ultrafiltration with a LB of 1 Hz) and therefore we considered that the effect of macromolecules was minimal.

We observed important differences among the various protocols tested in this work. The results were not always convergent and, to aid in the decision process, we gave more weight to the most relevant assessment parameters. In metabolomic studies, repeatability is one of the most important parameters indicating the reliability of the analytical method to identify potential markers (Wu et al., 2008; Markley et al., 2017). Regarding the two physical separation methods, skimming associated with ultrafiltration protocol provided better repeatability results with 72% of the buckets displaying a CV lower than 10%, compared to ultrafiltration protocol for which 65% of the buckets met the defined criteria. This could be due to a better elimination of lipid components with the skimming step, thus leading to a better selectivity, which improve repeatability. Regarding the two chemical-based protocols, methanol protocol provides better results than dichloromethane/methanol protocols. The latter is widely used for lipid isolation as the aqueous phase contains polar metabolites whereas non-polar metabolites stay in the organic phase. However, when applied to the milk matrix, no bucket had a CV inferior to 10% for the methanol/dichloromethane protocols. In addition, the repeatability for these two methods was poor compared to methanol alone or to skimming and ultrafiltration. Methanol/dichloromethane protocol is also time-consuming since it involves several steps. Moreover, the tested protocol, derived from (Folch et al., 1957), uses sodium chloride which can induce drifts in the chemical shifts and affect the NMR probe performance, impairing quantification (Bharti and Roy, 2012). Methanol protocol retains efficiently and selectively polar to mid-polar metabolites and precipitate proteins in one step (Want et al., 2006). Methanol protocol provided good repeatability results but also good recovery results as described in Table 2. The percentage of recovery ranged from 70 to 87%, compared to other protocols which barely reached 70%. This makes the methanol protocol simple and straightforward, which is important for high-throughput metabolomics studies. This protocol, together with skimming associated with ultrafiltration, showed the best repeatability that, as stated above, is likely the most important parameter. Skimming associated with ultrafiltration is a double-step process that eliminates lipids first, and then macromolecules (molecular weight greater than 10 kDa with the filter cut-off used in this work). Skimming associated with ultrafiltration is a physical separation protocol that appears to have a good resolution and with no interactions between a solvent and milk metabolites. This feature can represent an advantage, since no alteration of the metabolome may occur by potential artefactual reactions and alterations induced by the solvent. Moreover, physical separation does not involve evaporating steps, thus also avoiding losing volatile compounds. This method is often used for NMR milk metabolomics analysis. Nevertheless, skimming associated with ultrafiltration protocol showed a low yield compared to methanol and the presence of large amounts of proteins can lead to filter clogging. In addition, if we consider the advantage of applying a single protocol for performing multiplatform analyses, ultrafiltration is generally not used for LC-MS analysis because the higher sensitivity of LC-MS can lead to detection of filter contaminants (Tulipani et al., 2013).

Methanol protocol represented the best compromise in terms of spectral quality, repeatability, efficiency and extraction yield in our cow milk samples (Vuckovic, 2012). (Yang et al., 2016) successfully used methanol protocol and NMR analysis for differentiating milk metabolites from yak, camel, goat and cow. In contrast, (Wu et al., 2016) compared methanol and ultrafiltration for preparing human milk samples and concluded that ultrafiltration allowed a better identification and quantification of metabolites. This contrasting result might be due to differences in milk composition between species. Human breast milk contains 3 times less protein than cow's milk (Hernell, 2011). Removing excess proteins is essential to get a clear access to the metabolome, which explains why methanol precipitation seems more appropriate than ultrafiltration for cow milk analysis. An additional advantage of using methanol sample preparation for cow's milk metabolites NMR analysis is that it can also be easily transposed to MS analysis, which is interesting for a broad coverage untargeted metabolomics approach. The findings of this study may have applications for the analysis of milk from other animal species.

## 5 Conclusion

This study shows that methanol preparation is the best performing preparation method for NMR metabolomic analysis of cow milk samples. Milk is a biological matrix that can provide information on the nutrition, physiology and health status of the producing animal and metabolomics is a promising tool to study these aspects in dairy cows (Klein et al., 2012). The methanol preparation was chosen following a systematic comparison of all available milk preparation methods described in the literature. This sample preparation protocol was then successfully applied to discriminate the metabolite profile of milk from cows fed diets with minor differences in composition, highlighting the power and quality of the methanol sample preparation method selected in this work and its potential for experimental applications. A comparison of different milk preparation methods for NMR analysis was missing in the literature. The results of this study are useful for analytical laboratories working on cow milk metabolomics.

## Declarations

### Author contribution statement

Bénédict Yanibada, Cécile Canlet: Conceived and designed the experiments; Performed the experiments; Analyzed and interpreted the data; Contributed reagents, materials, analysis tools or data; Wrote the paper.

Hamid Boudra, Diego P. Morgavi: Conceived and designed the experiments; Analyzed and interpreted the data; Contributed reagents, materials, analysis tools or data; Wrote the paper.

Laurent Debrauwer: Conceived and designed the experiments; Wrote the paper.

Cécile Martin: Analyzed and interpreted the data; Contributed reagents, materials, analysis tools or data.

### Funding statement

Bénédict Yanibada was supported by a CIFRE (Industrial Agreements of Training by Research) PhD studentship funded by the ANRT (National Association of Research and Technology). This study is part of a collaborative project led by INRA and funded by 11 institutes and private companies: Adisseo France SAS (Antony, France), Agrial (Caen, France), APIS-GENE (Paris, France), Deltavit (Janzé, France), DSM Nutritional Products AG (Kaiseraugst, Switzerland), Institut de l'Elevage (Paris, France), Lallemand (Blagnac, France), Moy Park Beef Orléans (Fleury-les-Aubrais, France), Neovia (Saint Nolff, France), Techna France Nutrition (Couëron, France), and Valorex (Combourtillé, France). This work was carried out within the French Infrastructure for Metabolomics and Fluxomics (MetaboHUB ANR-11-INBS-0010). Milk samples used to test the selected protocol came from the PhD work of J. Guyader which was co-funded by Inra and Neovia.

### Competing interest statement

The authors declare no conflict of interest.

### Additional information

Data associated with this study has been deposited at the EMBL-EBI database MetaboLights under accession number MTBLS623 (https://www.ebi.ac.uk/metabolights/MTBLS623).

## Appendix A Supplementary data

Supplementary_Material_S2 Supplementary_Material_S2

Supplementary_Material_S3revised Supplementary_Material_S3revised
